# Assessment of cataract surgery outcomes at Jimma Medical Center, Jimma, Southwest Ethiopia

**DOI:** 10.3389/fopht.2025.1547898

**Published:** 2025-04-08

**Authors:** Amare Atoma Gelalcha, Sisay Bekele, Dagmawit Kifle, Wolela Mulatu, Edosa Kejela Keno, Wondu Reta Demissie

**Affiliations:** ^1^ Department of Ophthalmology, Faculty of Medical Sciences, Institute of Health, Jimma University, Jimma, Ethiopia; ^2^ Department of Anesthesiology, Faculty of Medical Sciences, Institute of Health, Jimma University, Jimma, Ethiopia; ^3^ Department of Biomedical Sciences, Faculty of Medical Sciences, Institute of Health, Jimma University, Jimma, Ethiopia

**Keywords:** cataract surgery outcomes, visual acuity test, physician quality reporting system measures, centers for Medicare and Medicaid services status, Ethiopia

## Abstract

**Background:**

Globally, cataract is the leading cause of blindness accounting for 51% and affecting approximately 39 million people. Visual rehabilitation is achieved through sight-restoring surgery.

**Objectives:**

The study aimed to assess the outcomes of cataract surgery that were performed by senior ophthalmologists and residents at Jimma Medical Center (JMC), Jimma, Southwest Ethiopia.

**Methods:**

An institutional-based cross-sectional study was conducted among 341 patients who underwent cataract surgery. The surgery outcomes were assessed using the Physician Quality Reporting System (PQRS) and WHO guidelines. The primary outcomes of the study were post-surgical visual acuity tests, complications within 30 days following surgery, and any additional procedures required. Finally, the outcome of cataract surgery was rated as good, borderline, or poor based on the post-surgical visual acuity test results according to WHO guidelines, and as good vision, no/mild, moderate, or severe visual impairment (VI), and blindness according to PQRS.

**Results:**

Out of the 341 cataract surgeries performed, 171 were operated by residents and 170 by senior ophthalmologists, respectively. The overall prevalence of cataract surgery outcomes based on PQRS guidelines for post-operative visual acuity tests showed good vision in 187 cases (54.8%), no/mild VI in 64 cases (18.8%), moderate VI in 46 cases (13.5%), severe VI in 12 cases (3.5%), and blindness in 32 (9.1%). According to the WHO classification, 253 cases (74.2%) had a good outcome, while 45 cases (13.25%) had a borderline outcome and 43 cases (12.6%) had a poor outcome. The prevalence of cataract surgery outcomes varied among healthcare professionals performing the surgeries. Less than 9.7% of patients required re-surgery within the first month of operation due to complications.

**Conclusion:**

In summary, 54.8% of the patients achieved good vision with an acuity test result of 6/12 or better. This finding meets the minimum Medicare PQRS measure value of ≥50% for both professionals. The overall outcome of cataract surgery showed a statistically significant difference between residents and senior ophthalmologists who performed the procedures.

## Introduction

A cataract is defined as any opacity of the crystalline lens of the eye (1). It is the leading cause of blindness globally, particularly in developing countries, accounting for 51% of the 39 million blind people worldwide, where 90% of them reside in low- and middle-income countries (2–5).

Cataracts are typically associated with old age, but cataracts can develop in all age groups due to various causes such as congenital factors, trauma, systemic diseases, intraocular diseases like uveitis, or some medications like steroids (6–8). Ultimately, the risk factors of cataracts are genetic factors, age, smoking, high alcohol consumption, lower socioeconomic status, and/or lower education, poor nutrition, metabolic and systemic diseases, severe diarrhea, acidosis and dehydration, environmental factors (exposure to sunlight and radiation), host factors, and medications (9–12).

Cataracts usually develop in both eyes, although they may progress at different rates. It can take anywhere from a few months to several years for a cataract to form and interfere with vision. Nearly everyone has some cataract formation by age 70 (13–16).

Cataracts impose a major burden on the individual, family, society, and the country as a whole, with its treatment being costly (17–22).

While there are no proven methods for preventing cataracts, surgery is the primary treatment option. Indications for cataract surgery include visual impairment (VA of less than 3/60 in Ethiopia) or when vision interferes with daily activities (23–25). The medical indications of cataract surgery are diabetic retinopathy (Media Clarity for Rx and Phacomorphic glaucoma), the health of the eye, a dislocated lens into the anterior chamber, cosmetic indication, and restoration of a black pupil in a blind eye (26–29).

Cataract surgery is one of the most successful surgical procedures performed today. Over 95% of people who underwent surgery reported significant improvement in their vision. Cataract surgery is quick and gives immediate outcomes. Cataracts can be removed at any time when the individual feels that it has interfered with the daily activity/life. Different types of cataract surgery are currently done [phacoemulsification and intra-capsular cataract extraction (ICCE)]. Visual rehabilitation is made through sight-restoring surgery. In recent years, the number of people who undergo cataract surgery has increased rapidly, and it has become the most frequently performed cost-effective surgical procedure throughout the world (30–37).

The outcome of cataract surgery is crucial, with measurements based on post-operative visual acuity, functional ability, quality of life, and economic rehabilitation (38–41). The patients’ visual satisfaction, vision-related quality of life, ability to function in daily activities, and their overall productivity mainly depend on the visual outcome (42).

World Health Organization recommends the outcome of cataract surgery based on the level of best-corrected visual acuity (BCVA) where the poor outcome (BCVA <6/60) or borderline (BCVA <6/18) after cataract surgery should not be >10%–20% (43). Furthermore, several studies revealed that approximately 30%–50% of cataract-operated eyes cannot see, 6/60 (=20/200; 0.1), with the correction available to them, and the visual outcome of cataract surgery does not meet the individual’s daily visual demand due to concurrent sight-impairing eye diseases, early surgical complications, inadequate optical correction, or long-term complications (44–50).

In addition to WHO BCVA criteria, currently, other techniques are applied to assess the outcome of cataract surgery [the physician quality reporting system (PQRS) and the Centers for Medicare and Medicaid Services (CMS)] to evaluate the quality of care delivered by physicians as the objective indicator of performance. To date, there are no studies evaluating the cataract surgery outcomes that were done by senior and resident ophthalmologists by employing multiple outcome indicators in the setting. Thus, the present study aimed to assess the cataract surgery outcomes that were performed by senior and resident ophthalmologists (hypothesized if there is a difference in outcome among professional skills) by employing multiple outcome indicators (WHO, PQRS, and CMS guidelines).

## Methods

A hospital-based cross-sectional study was conducted from April to July 2022 at the Ophthalmology Clinic of Jimma Medical Center (JMC), located in Jimma town, approximately 354 km from the capital city of the country, Addis Ababa (Finfine) to the Southwest direction.

JMC is the only tertiary, referral, and specialized hospital in Southwest Ethiopia providing services to a catchment population of approximately 20 million population. Among the medical departments at JMC, the ophthalmology/eye clinic is of paramount importance. The eye clinic of JMC serves the entire population of the Southwest region of the country with a total staff of 75 including 8 senior ophthalmologists, 16 residents from first to fourth years, 6 optometrists, 4 ophthalmic nurses, 25 general nurses, and other staff.

The study was conducted among patients with cataracts who attended follow-up for post-cataract surgery at JMC’s eye clinic. All cataract patients who underwent cataract surgery by senior surgeons and third and fourth year residents were included in the study. Patients’ clinical conditions were evaluated by reviewing their medical cards, taking history, physical examination, and laboratory investigation. Accordingly, cataract patients were strictly evaluated pre-operatively before cataract surgery, and finally, approximately 341 from a total of 1,600 cataract patients operated from 2020 to 2022 were included in the study, while cataract patients with other co-morbid eye diseases (uveitis, amblyopia, cataract secondary to ocular disorders, traumatic cataracts, cloudy cornea, corneal opacity, corneal edema, degenerative disorders of the globe, diabetic macular edema, diabetic retinopathy, disorders of the optic nerve, optic atrophy, optic neuritis, glaucoma, glaucoma associated with congenital anomalies and dystrophies, injury to the optic nerve and pathways, retinal detachment with retinal defect, retinal vascular occlusion, retinopathy of prematurity, and pediatric cataract) were excluded. Some cataract surgeries that required devices or techniques not generally used in routine cataract surgery like suture support for IOL, primary posterior capsulotomy for pediatric cataract, post-phacoemulsification type of cataract surgery, and traumatic cataract combined surgery cases were also excluded.

The outcomes of cataract surgery were assessed based on best-corrected visual acuity (BCVA), pre- and post-operative complications following established criteria from PQRS, CMS, and WHO as good, borderline, and poor.

The outcome of cataract surgery as per PQRS was rated as good vision if a threshold of reimbursable quality of care at 50% was established by assessing the following: a) visual acuity of better than 6/12 following cataract surgery, b) presence or absence of complications within 30 days following cataract surgery requiring additional surgical procedures, c) improvement in patient’s visual function within 90 days following cataract surgery, and d) patient satisfaction within 90 days following cataract surgery (44).

BCVA as a visual acuity test was done for all patients after all correctable causes of decreased vision were corrected like refraction. Post-operative visual acuity test after cataract surgery was also measured, and the patient may be refracted.

The outcome of cataract surgery as per WHO guidelines was rated as follows: a) good outcome, if post-operative BCVA was ≥6/18 and >80%–90% from the total cataract surgeries done; b) borderline outcome, if post-operative BCVA <6/18–6/60 and <5%–10% from the total cataract surgeries done; and c) poor outcome, if post-operative BCVA <6/60 and <5% from the total cataract surgeries done (43).

Visual acuity test was measured using the Snellen E-chart by ophthalmic nurses. Other detailed eye examinations like evaluation for ocular adnexal, anterior segment, posterior segment, and dilated funds examination by Slit lamp was done, and any abnormalities were documented and treated. For instance, those patients who have post-operative complications like posterior capsule opacification were treated by YAG-laser capsulotomy, and those who need referral were referred to appropriate referral clinics and better setup for better management. Refraction of post-operative follow-up patients was done by optometrists, in which almost all study participants were refracted after at least 6 weeks post-operatively. Ocular biometric parameters such as axial length, anterior chamber depth, and radius of corneal curvature were measured by automated keratorefractometer (Retinomax) and Sonomed A-Scan (Model 300AP) using contact applanation method. The power of intraocular lens needed for each cataract eyes was calculated using SRK-T formula (51).

The data were cleaned, coded, and entered into Epidata (4.6.1) and finally exported to SPSS (25) for further analysis. Descriptive statistics like frequency and percentage were computed to show the pictures of data, and the finding was reported by narration, tables, and figures.

The study was approved by the ethical review board committee of Jimma University, and a letter of support was also collected from JMC and the eye clinic to conduct study. Both verbal and written consents were taken from the participants, and each study respondent was informed about the research and given a free will to take part in the study. The patients’ information was kept confidential.

## Results

### Socio-demographic and clinical profiles of cataract patients

From a total of 1,600 cataract surgeries done from 2020 to 2022, approximately 254 patients with 341 operated eyes were finally included in the study. Among those, 167 (65.74%) patients have had been operated for one eye; almost equally (171 and 170 patients) were done by residents and senior ophthalmologists, respectively. The mean age of patients were 64 ± 12 years, ranged from 16 to 96 years where majority of them (59.8%) belonged the age groups of 55–74 years. A majority of the respondents were men (56.9%) with a male-to-female ratio of 1.27: 1 (**Table 1**).

**Table 1:** Socio-demographic characteristics of patients with cataract who underwent cataract surgery in JMC’s eye clinic from 2020 to 2022.

Variables	Category	Frequency	Percentage
Age in years	16–34	18	5.3%
	35–54	48	14.1%
	55–74	204	59.8%
	75–94	70	20.5%
	>95	1	0.3%
Sex	Male	194	56.9%
	Female	147	43.1%
Place of residency	Rural	242	71.0%
	Urban	99	29.0%
Educational status	No formal education	286	83.9%
	Literate	55	16.1%

A majority of the participants have no chronic medical illness; only 16% of them have a history of chronic medical illness. Hypertension was the major one, which constitutes 9.4%, followed by diabetes mellitus and bronchial asthma constituting 2.1% and 1.5%, respectively. There were also few patients with different types of medical illness like cardiac disease, chronic kidney disease, goiter, parkinsonism, stroke, spinal kyphosis, and major depressive disorder. However, all those patients with the abovementioned medical illness have no history of known ocular involvements. After cataract extraction was done and upon funds examination, only two of those patients with known hypertension have hypertensive retinopathy, and from diabetic patients, only one patient with bilateral diabetic retinopathy of severe stage was detected.

Only four (1.2%) patients have had history of previous ocular illness. Two of the patients had history of trachoma that was diagnosed and treated previously, one patient had history of allergic eye diseases, and the other patient had history of ocular infection associated with redness and eye discharge treated previously. All of these patients did not have any form of chronic sequels of these disease entities upon ocular examination.

Of all the study participants, only five (1.2%) patients had history of previous non-cataract ocular surgery: three underwent surgery for trachoma that was tarsotomy from patient’s charts documentation, one had surgery due to epiphora but no documentation specifically about the type of surgery done for him, and one had history of eyelid surgery for lid correction (**Table 2**).

**Table 2:** Pre-operative ocular and medical conditions of patients with cataract who underwent cataract surgery in JMC’s eye clinic from 2020 to 2022.

Variables	Category	Frequency	Percentage
Co-morbidity	No	286	84.1%
	Hypertension	32	9.4%
	Diabetes mellitus	7	2.1%
	Asthmatic	5	1.5%
	Autoimmune diseases	2	0.6%
	Cardiac	3	0.9%
	Chronic kidney disease	2	0.6%
	Stroke	1	0.3%
	Goiter	2	0.6%
Patient previous ocular illness	No	336	98.8%
	Yes	4	1.2%
Non-cataract ocular surgery	No	336	98.8%
	Yes	4	1.2%
Pre-operative complicated	No	260	76.5%
	Pseudoexfoliation	57	16.8%
	Sub-luxated lens	10	2.9%
	High intra-ocular pressure	1	0.3%
	Hypotonic	1	0.3%
	Strabismus	9	2.6%
	Others	2	0.6%

The study participants were classified into two groups for comparison purposes; those operated by residents were 171 (50.147%). There was no statistically significant association of intra-operative complication with the level of surgeons with p-value >0.05.

A majority of them have been followed up to 5–6 weeks post-operatively, which constitutes 23.8%. One-third of the patients used their medication for 2 months. Only 4.1% of the study participants used medication for <2 weeks post-operatively. A majority of the study participants had improvements of their vision and their functioning capacity post-operatively, which constitute 93.3%. Delivery of health education post-operatively to the patient was not adequate. Only one-fourth of the study participants had health education on how to use the medication, what should they do and not do, and when to come back before appointment. Most of the study participants have no any eye complaints post-operatively when asked, which constitutes 70.4% of the patients. There were three patients who complained of ptosis, metamorphopsia, and crocodile tearing. The prevalence of post-operative complication and re-operation was 26.7% and 9.6%, respectively. In this study, the correct VAs used for this study were those taken after 6 week post-operative and beyond the post-operative period. Before refraction, only 17.3% of the operated patients have had good vision. A majority of the patients were not refracted post-operative, even though they have poor vision and have been for more than 3 months up to a year and beyond. Only 13.5% of the patients were refracted. Almost all study participants who passed the sixth week post-operative were refracted, and there was a great significant improvement of the patients’ VA after refraction, which was two-thirds of the cases. After post-operative refraction and those who have vision improvements and who need spectacle wear, a majority (78.06%) of the patients have been using eyeglasses. The majority of those patients who refused spectacle to improve their vision have financial problems, approximately 82.7% (**Table 3**).

**Table 3:** Post-operative conditions and medication use of patients with cataracts who underwent cataract surgery in JMC’s eye clinic from 2020 to 2022.

Variables	Category	Frequency	Percentage
Post-operative complications	No	250	73.3%
	Yes	91	26.7%
Post-operative medication use (in weeks)	1–2	14	4.1%
	3–4	55	16.2%
	5–6	80	23.5%
	7–8	112	32.9%
	9–12	18	5.3%
	>12	61	17.9%
Post-operative patient’s Feeling	Improved	317	93.2%
	Same	23	6.8%
Post-operative health education	No	255	75.0%
	Yes	85	25.0%
Re-operation	No	308	90.4%
	Yes	33	9.6%
Post-operative refraction	No	295	86.5%
	Yes	46	13.5%

### Outcomes of the cataract surgery

BCVA of those operated by residents had better outcomes than those operated by senior ophthalmologists. The causes of less visual outcome among these cases operated by the senior surgeons were not identified in this study. However, slightly more pre-operative complicated cases were done by the senior ophthalmologists. Of all the study participants, only 23.7% of cases had pre-operative complicated cases where the majority of them (55.56%) were operated by senior ophthalmologists.

In a nutshell, the outcome of cataract surgery as per PQRS was rated as follows: a) good vision was 63.16% vs. 46.47% among cataract surgeries done by residents and senior ophthalmologists, respectively; b) no or mild VI was 18.71% vs. 20% among cataract surgeries done by residents and senior ophthalmologists, respectively; c) moderate VI was 8.77% vs. 17.65% among cataract surgeries done by residents and senior ophthalmologists, respectively; d) severe VI was 3.51% vs. 3.53% among cataract surgery by residents and senior ophthalmologists, respectively; and e) post-operative blindness was 5.85% vs. 12.35% among cataract surgery done by residents and senior ophthalmologists, respectively (**Table 4**).

**Table 4:** Outcome of cataract surgery (PQRS) by residents vs. senior surgeons among patients with cataracts in JMC’s eye clinic from 2020 to 2022.

PQRS outcome	Category	Done by residents; No. (%)	Done by senior surgeons; No. (%)	Outcome of all patients; No. (%)	p-value
1	Good vision, 6/6–6/12	108 (63.16)	79 (46.47)	187 (54.8)	0.04
2	No/mild VI, <6/12–6/18	32 (18.71)	34 (20)	64 (18.8)	
3	Moderate VI <6/18–6/60	15 (8.77)	30 (17.65)	46 (13.5)	
4	Severe VI, <6/60–3/60	6 (3.51)	6 (3.51)	12 (3.5)	
5	Blindness, <3/60	10 (5.85)	21 (12.35)	32 (9.4)	

When assessed by the WHO outcome of the cataract surgery approach, all study participants had 74.2% good outcomes, whereas the prevalence of good cataract surgery outcomes was 81.88% and 66.5% among surgeries done by residents and senior ophthalmologists, respectively. The prevalence of borderline and poor outcome is detailed in **Table 5** for cataract surgery outcomes.

**Table 5:** Outcome of cataract surgery (WHO) by residents vs. senior surgeons among patients with cataract in JMC’s eye clinic from 2020 to 2022.

BCVA Outcome (WHO)	By residents; No. (%)	By senior surgeons; No. (%)	Total cases; No. (%)	p-value
Good outcome (6/6–6/18	140 (81.88)	113 (66.5)	253 (74.2)	0.05
Borderline outcome (<6/18–6/60)	15 (8.78)	30 (17.65)	45 (13.2)	
Poor outcome (<6/60)	16 (9.34)	27 (15.85)	43 (12.6)	

## Discussion

The present study aimed to assess the cataract surgery outcomes at an academic teaching institution using the Medicare PQRS criteria and based on WHO guidelines of cataract surgery outcomes. The data show that residents’ cases in more than 50% of the surgeries exceeded Medicare PQRS criteria but were below the WHO guideline cutoff points. This study demonstrated that residents were able to meet Medicare PQRS criteria for cataract surgery outcomes.

When the outcomes of cataract surgery operated by trainers and senior surgeons were compared based on BCVA, those operated by residents had better outcomes than those operated by senior ophthalmologists relatively. Approximately 54.84% of all patients had good vision, 6/12 and better, so that it meets the minimum PQRS threshold, which requires at least more than 50%, and the residents’ cases with good vision were 63.16%, fulfilling the minimum requirement. However, the senior ophthalmologists’ cases with good vision were 46.47%, which did not fulfill minimum requirements.

The present finding for the prevalence of good vision outcome of 54.84% was inferior when compared with one study done at the Massachusetts Eye and Ear, which reported the prevalence of good vision (achieved 6/12 vision) of 98.95% in all, 98.9% of residents, and 99.0% of senior ophthalmologists’ cases (44). The reasons for this difference might be the type of surgery in the above study, which was phacoimmulsification type, while it was MSICS in our study; the quality of hospitals set up with the availability of equipment; the quality of training delivery for trainers; and patients awareness on post-operative care.

Overall, only 74.2% of the patients had good outcomes, and it was against the WHO guideline (should be 80%–90%). Of the residents’ cases, 81.88% achieved good outcomes and met at least the targets, but those operated by senior ophthalmologists who achieved good outcomes were only 66.5%, which is below WHO standards requirements (43).

The present finding of 74.2% of good vision outcome for all patients was higher than that of a study done at Gondar University Hospital, which reported a prevalence of good visual outcome of 26.6% and 28.4% (52, 53); Goro District, Central Ethiopia, in which good visual outcome was obtained in 23.7% (54); Gujarat, India, 71.4% (55); Adelaide, South Australia, 41.2% (56); Southern China, 62.2% (57, 58); Kuwait, 54% (59); Jimma University, Ethiopia, 70.4% (60); and Saint Paul’s Millenium Medical College Hospita Addis Ababa, 68.5% (61). However, this finding was slightly lower than the one done at Ibadan, Nigeria (78.8%) (62, 63). The disparity in the finding could be due to multiple attributes.

The causes of less visual outcome among these cases operated by the senior surgeons were not clearly identified in this study. However, slightly more pre-operative complicated cases were done by the senior ophthalmologists. From the all study participants, only 23.7% of cases had pre-operative complicated cases where majority of them (55.55%) were operated by senior surgeons. There was no statistically significant difference in proportion of pre-operatively complicated cases that were operated by senior ophthalmologists vs. residents. The possible explanation for the better outcome of the residents’ cases might be that most of the residents’ cases were clear cataract, under supervision of senior attending surgeons, with early intra-operative consultations, and they might be assisted by senior ophthalmologists on needs and they operated few patients daily in a careful and slow manner, while senior surgeons operated many patients with speed to complete the patients scheduled.

Only <9.4% of the all patients return back to the operating room within the first month of operation for complication and also varies among professionals, 8.2% and 10.6% among residents’ and senior ophthalmologists’ cases. This finding was much higher than the finding of one study done at the Massachusetts Eye and Ear that reported 0.65% and 0.64% re-operated cases by residents and senior ophthalmologists, respectively (44). This might be explained by the difference on the type of surgery, facility of the hospital’s setup, and quality of level of training.

A majority of the cases had no intra-operative complication; only 14.4% of the case had at least one of the following intra-operative complications. The most common complication was PC tear + vitreous loss, 6.2%; PC tear + vitreous loss + aphakia, 4.1%; and only PC tears, 1.5%. When compared with another study done at New South Wales, Australia, the intra-operative complications were slightly higher in our study; only11.9% were complicated intra-operatively. The most common intra-operative complications were posterior capsular tear, with or without vitreous loss in 3.4%, anterior capsular tear in 2.0%, and dropped nucleus in 0.3%.

The complications that occurred also varied among cases done by trainers/residents (16.4%) and senior ophthalmologists (12.3%) and those also supported by other studies (44, 63, 64).

Most of the causes of post-operative poor vision were surgery-related complications (intra- and post-operative).

In more than four-fifths of the operated eyes, biometry was done. The correctly calculated IOL was implanted for only 57.73% of the operated eyes. Most of the patients were not refracted post-operatively before being interviewed for the questionnaire, even though they have had poor vision and have been coming for more than 3 months up to a year and beyond. Only 13.5% of the patients were refracted. Almost all study participants were refracted (those after 6 weeks post-operative), and this study concluded that as there was a great improvement of the patients’ VA after refraction, approximately 69.5% of the patients have at least more than one visual acquit test line improvement on their vision. In comparison with the other literature studies done at Harvard, Australia, and Ghana in which all patients were refracted for their BCVA (44, 63, 64), this value shows that there are very poor habits of patients post-operative refraction after 6 weeks post-operative to find their BCVA in this hospital. Thus, those health professionals working on post-operative follow-up should be oriented on the need to refract all patients.

## Limitations of the study

This study was conducted in only one ophthalmic teaching center, so it might not be representative of all ophthalmic training centers in the country. The difference in cataract surgery outcomes among senior ophthalmologists and residents might be due to the limitation of the study in matching the operated cases with higher chances of complications and co-morbidities.

## Conclusion and recommendation

Overall, more than half (54.8%) of the patients who underwent cataract surgery had good vision, 6/12 and better, thus meeting the minimum Medicare PQRS measure threshold, which requires at least more than 50%; the resident’s cases also fulfill the minimum requirement, in which 63% had good vision of 6/12 and better. However, cases done by senior ophthalmologists had only 46% of good vision, which is below 50%, the PQRS requirement. This study shows that the senior ophthalmologist’s case outcomes were relatively less than that of the trainers; this needs an explanation that requires another study or research.

Based on the WHO guideline outcome, only 74.2% of the patients had good outcome, so this figure was below the WHO standard. From those operated by residents, 81.88% achieved good outcome that meets at least the targets, but those operated by senior ophthalmologists only achieve 66.5% of good outcome (below WHO standards requirements).

The prevalence of poor outcome of cataract surgery was 12.6%, which was significantly below the WHO requirements. The rate of poor outcome also varies among residents and senior ophthalmologists (9.34% and 15.85%), respectively, and below WHO standard.

Overall, only 9.6% of the study participants were re-operated for complications within a month of surgery. A majority of these cases were re-operated by the senior surgeons (10.6%).

## Data Availability

The original contributions presented in the study are included in the article/supplementary material. Further inquiries can be directed to the corresponding author.
